# Parsonage-Turner Syndrome After COVID-19 Vaccination in a Child

**DOI:** 10.5435/JAAOSGlobal-D-22-00156

**Published:** 2023-03-09

**Authors:** Elisa Masnou Cassart, Dolores Rolan Vilas, Ryo Abe, J. M. Cavanilles-Walker

**Affiliations:** From the Department of Orthopaedic Surgery and Traumatology. Hospital Germans Trias i Pujol, Badalona (Dr. Cassart, and Dr. Cavanilles-Walker); the Neurology Service, Department of Neuroscience, Hospital Germans Trias i Pujol, Badalona (Dr. Vilas); and the Centro de Rehabilitación Funcional Montigalà, Badalona, Barcelona, Spain (Abe).

## Abstract

Parsonage-Turner syndrome (PTS) is a peripheral neuropathy involving the brachial plexus very rare in childhood. To date, no cases of PTS after COVID-19 vaccination have been reported in children. We report a case of a 15-year-old boy affected by PTS after the second dose of the BNT162b2 (Comirnaty, Pfizer-BioNTech) COVID-19 vaccine.

Parsonage-Turner syndrome (PTS) is a rare peripheral neuropathy involving the brachial plexus characterized by sudden onset pain affecting the upper extremity associated with muscle weakness. With an estimated incidence of 1.64 cases per 100,000 people,^1^ PTS usually develops in people aged between 20 and 60 years, being very rare in children.

The diagnosis is based on clinical history, physical examination, electrophysiological study, and exclusion of other pathologies. The clinical picture of PTS is very diverse because a large variety of nerves can be affected. Muscle paresis commonly manifests as scapula alata, secondary to paresis of the muscles involved in scapular fixation.^2^ Pain is the first symptom in 90% of patients, being the location of pain variable, but in most cases radiated from the cervical spine or shoulder region into the arm.^1^ Electrodiagnostic testing is widely used to diagnose PTS. Abnormal sensory potentials, lack of denervation potentials, and abnormal conduction velocities are the most frequent findings.

The etiology and pathophysiology of PTS still remain unclear.^3^ Recent viral infections, such as herpes simplex virus, Epstein-Barr virus, cytomegalovirus, HIV, hepatitis B virus, and parvovirus B19 infections, have been reported as the probable trigger in 25% of patients with PTS.^4-6^ Vaccination has been described to precede PTS in 4.3% to 15.5% of cases.^1,5^ To date, only a few cases of PTS after COVID-19 vaccination have been reported, none of them in the childhood.^7-15^

In this study, we report a case of a child affected by PTS after the second dose of COVID-19 vaccination.

## Case

A 15-year-old boy presented to the clinic because of acute pain and functional impotence in his left shoulder that started 1 week before. Physical examination revealed reduced range of motion for shoulder flexion and abduction because of weakness and pain. He had full flexion and extension of the wrist and fingers as well as hand interosseous muscles. Four weeks earlier, he had received the second dose of the BNT162b2 (Comirnaty, Pfizer-BioNTech) coronavirus disease (COVID-19) vaccine. Since the administration of the first dose, 3 weeks before the second one, he suffered from hypoesthesia in his left shoulder. His medical history was normal. No previous vaccine reactions were recorded. The pain was partially controlled after placing the arm in a sling together with oral nonsteroid anti-inflammatory drugs. After 2 weeks of treatment, the pain improved, but severe paresis in the left shoulder, mainly in the elevation and abduction involving the deltoid, and also in the infraspinatus muscle, along with hypoesthesia in the lateral aspect of the shoulder, after the distribution of the axillary nerve, still persisted. Cervical spine and left shoulder MRI performed 2 weeks after the onset of symptoms were normal. The electrophysiological study, performed 4 weeks after the onset of symptoms, showed a severe axonal loss of the left axillar nerve with fibrillation and positive spontaneous waves in the deltoid muscle, suggesting a severe acute denervation.

The clinical features, the recent vaccination, and the electrophysiological findings lead us to the diagnosis of a PTS. The patient began an intensive rehabilitation program, 1 hour every day, consistent in ultrasonography and electrical stimulation therapy for 20 minutes, along with passive range of movement and active exercises. At the beginning, passive scapular mobilization and pectoralis stretching as well as isometric lower trapezius and external rotation were done. Progressively, shoulder elevation with pulley and active scapular recentering were introduced. Finally, eccentric exercise proprioception and strength exercises with rubber bands were implemented. After 6 months, the patient recovered a full shoulder range of movement but still was showing persistent atrophy in the anterior deltoid muscle and partial weakness of the deltoid 4+/5, infraspinatus 3+/5, and supraspinatus 4/5, with persistent hypoesthesia in the lateral aspect of the upper arm. A new electrophysiological study revealed a slight spontaneous electrical activity with polyphasic elements, along with focal axonal loss in the deltoid muscle. The presence of abundant polyphasic elements of reinnervation stood out. The neurography of the left suprascapular and axillary nerves exhibited severely altered responses in the left axillary nerve with focal and axonal blockade. At the 1-year follow-up, the patient recovered full range of motion and strength as well as axillary nerve sensitivity.

## Discussion

In this study, we report the first case of a child-onset PTS after COVID-19 vaccination. Although the etiology of PTS is still unclear, an immune-mediated inflammatory reaction against the affected nerve in genetically predisposed patients triggered by multiple factors has been suggested. SARS-CoV-2 infection has been described as a possible initiating event for PTS.^16-21^

Regarding vaccination as a possible initiating factor, PTS has been linked most recurrently to the administration of tetanus, human papilloma virus, influenza, and encephalitis vaccines.^1,4,5^ In children, it has also been described after immunization for diphtheria, tetanus, and pertussis.^5^ To date, few cases have been described after COVID-19 vaccination,^7-15^ none of them in the children. The time from vaccination until the onset of PTS symptoms has been described around 28 days.^22^ Specifically, after COVID-19 vaccination, it has been reported to be between 13 hours and 8 weeks.^10,13^ Our patient reported the onset of symptoms 4 weeks after vaccine administration suggesting a correlation between PTS and the vaccine administration in the absence of other related factors.

The diagnosis of PTS is mainly based on the clinical presentation and the electrophysiological findings. A rapid onset of severe pain in the shoulder and arm, followed by weakness of the muscles in the affected areas should make us to suspect this entity. Neurophysiologic studies are the best modality to confirm the diagnosis. Abnormal sensory potentials, lack of denervation potentials, and abnormal conduction velocities are the most frequent findings. In general, neurophysiologic studies should be done 2 to 3 weeks after the onset of symptoms because there is no degenerative action potential in the acute stage. In our case, although different shoulder muscles were paretic, we could only observe EMG abnormalities in the axillar nerve. This could be explained, at least partly, because the deltoid muscle was the most affected one. In addition, other possible pathologies which could explained the clinical picture of our patient, such as cervical spine radiculopathy or entrapment neuropathy, were ruled out. The MRI could be useful to rule out other etiologies and to depict the sequelae of skeletal muscle denervation. Intramuscular edema usually appears after a few weeks, and in advanced stages, muscle atrophy and fatty infiltration might be seen.

The current treatment recommendations for PTS include a combination of analgesics, nonsteroid anti-inflammatory drugs, corticosteroids, and anticonvulsants. Physical therapy must be done early to achieve better final functional results.^1,23^ About 70% of adult patients may present residual paresis,^1^ while 63% of children achieve full recovery.^23^

Other post vaccine-related inflammatory responses apart from PTS are well known. Shoulder injury related to vaccine administration (SIRVA) is a recognized complication presenting with significant shoulder pain and stiffness. It is thought to occur because of improper administration of the vaccine into the subdeltoid bursa or shoulder joint leading to an inflammatory cascade leading to conditions such as adhesive capsulitis, subacromial bursitis, rotator cuff tendinitis or tears, or even subcortical bone osteitis.^4^ Although it has been reported more frequently with influenza tetanus, pneumococcus, and papillomavirus vaccinations, the first case of SIRVA after COVID-19 vaccination was reported in April 2021 by Cantarelli et al.^24^ Although our patient initially had pain, the fact that the symptoms progressed in the form of painless paresis along with the absence of remarkable findings around the shoulder on the MRI makes it possible for us to reasonably rule out SIRVA.

Although the exact cause of PTS is still unknown and is not possible to establish a causative connection between this case of PTS and the administration of the COVID-19 vaccine, the close association between the two events in the absence of other potential causes makes our diagnosis feasible. A total of 11000.3 million vaccine doses have been administrated in the world by April 1, 2022, with more than 58% of the world population fully vaccinated.^25^ Considering these data, additional cases of PTS after COVID-19 vaccination are to be expected in the near future calling attention on its early diagnosis, which is essential for a suitable management and prognosis.
